# Effects of different initial bundle tensioning strategies on the outcome of double-bundle ACL reconstruction: a cohort study

**DOI:** 10.1186/1758-2555-3-15

**Published:** 2011-07-28

**Authors:** Takeshi Muneta, Hideyuki Koga, Young-Jin Ju, Kazuyoshi Yagishita, Ichiro Sekiya

**Affiliations:** 1 Department of Orthopaedic Surgery, Tokyo Medical and Dental University Hospital, Tokyo, Japan

## Abstract

**Background:**

This study was performed to investigate the effects of different strategies and initial tension applied to each one of the bundles, antero-medial (AM) and postero-lateral (PL), on clinical outcome in double bundle (DB) ACL reconstruction.

**Methods:**

One hundred fifty-one primary unilateral DB ACL reconstructions performed by a single surgeon from 1994 through 2002 were included in the study with a follow-up of at least 24 months. They were divided in the following 3 groups: Group I - Higher initial tension applied manually in the AM bundle compared to PL. II - Higher tension applied in the PL bundle compared to AM. III - The 2 bundles were attempted to be equally tensioned. All fixations were performed in 30 degrees of flexion. Group I = 59 patients, group II = 53 patients and group III = 39 patients. The groups had no statistical differences concerning demographic distribution. Clinical outcome was retrospectively evaluated by use of knee range of motion, manual knee laxity tests, KT-1000, Lysholm knee scale, subjective recovery scale and sports performance recovery scale. The differences of data were analyzed among the three groups.

**Results:**

Group I showed a significant extension deficit compared with groups II and III. ANOVA revealed a significant difference of anterior laxity measured by the KT-1000 (average KT difference of 2.1, 2.1 and 1.2 mm in Group I, II and III, respectively). A statistical difference was found among the three groups regarding subjective and sports performance recovery scales with Group II showing higher scores in recovery than Group I.

**Conclusions:**

The current clinical study does not recommend manual maximum of initial tension applied to the anteromedial or posterolateral bundles with graft tension imbalance at 30 degrees of flexion in double-bundle ACL reconstruction to achieve a better clinical outcome.

## Background

Recently, several clinical studies have addressed the advantages of double-bundle (DB) anterior cruciate ligament (ACL) reconstruction using medial hamstring tendons in achieving better anterior and rotational stability compared with single-bundle (SB) reconstruction [1-3]. One of the features of DB ACL reconstruction is the possibility of fixing each bundle, antero-medial (AM) and postero-lateral (PL), separately with an optional load.

A number of in-vitro cadaveric studies have analysed the effects of initial tensioning on kinematic and stability change of the knee for achieving better clinical results [4-6]. These cadaveric studies sounded alarm against too much tension of the PL bundle in DB ACL reconstruction. Murray PJ, et. al investigated the two protocols of each graft tensioned to 22 N in human cadaveric anatomic DB reconstruction. The posterolateral (PL) bundle tensioned at 15 degrees and anteromedial (AM) bundle at 45 degrees protocol led to excessive tension in the AM bundle in full extension [7].

The biologic effects of initial tension difference have been investigated in a dog ACL reconstruction model using a bone-patellar tendon-bone (BPTB) graft. The results showed a better healing process of the graft in the 1 N initial tension group than the 20 N initial tension group 3 months after surgery [8].

Several clinical studies have investigated the effect of initial graft tension on clinical outcome. Yasuda et al. showed an inferior clinical outcome for the group with initial 20 N of tension in comparison to the 40 N and 80 N groups in SB hamstring tendon reconstruction with regard to the objective knee stability [9]. Yoshiya et al. found no significant difference objective stability as well as subjectively between 25 N and 50 N as initial tension for BPTB graft in ACL reconstruction [10]. A randomized study with BPTB reconstruction has concluded that a graft tension of 45 N was not sufficient for restoring knee objective stability compared with 90 N [11].

In clinical studies of anatomic DB ACL reconstruction, too much tension of the PL bundle has been raised as a potential problem causing extension deficit or decrease of the PL bundle function [12,13]. The high tension in the PL bundle would be caused by its tension pattern as well as the high initial force setting for the PL bundle. Recently, good clinical outcome has been reported at 2 years postoperatively in the anatomic double-bundle ACL reconstruction with 20 N of initial tension [14]. However, there has been no report investigating the effects of initial graft setting in comparative studies in a DB reconstruction method.

The purpose of this study is to investigate the clinical outcome of three groups with different strategies of initial graft force setting during a DB ACL reconstruction. The hypothesis of the study is that both AM and PL bundles should be equally tensioned at 30 degrees of flexion as initial force setting to achieve better knee stability.

## Methods

Written informed consent was obtained from the patient for publication of the study. A copy of the written consent is available for review by the Editor-in-Chief of this journal if necessary.

### Patients

A cohort of 153 primary unilateral DB ACL reconstructions using autogenous medial hamstring tendons by a single surgeon (TM) from December 1994 through October 2002 was followed up with data collection of at least 24 months, and retrospectively analysed for clinical data. The study consisted of three cohorts as follows: Group I (AM bundle highly tensioned group), Group II (PL bundle highly tensioned group) and Group III (AM and PL bundles equally-tensioned group) according to the graft fixation procedure of each double bundle during surgery.

In Group I, the PL bundle was first fixed to the post screw at 30 degrees flexion with manual pull, and then the AM bundle was fixed to the post screw at 30 degrees flexion with manual maximum pull. In Group I, the initial tension of the AM bundle was intentionally higher than that of the PL bundle in every case at 30 degrees flexion which was arthroscopically confirmed by probing after fixation. Group I was performed from December 1994 to September 1996 in 73 cases by TM.

In Group II, the AM bundle was first fixed to the post screw at 30 degrees flexion with manual pull, and then the PL bundle was fixed to the post screw at 30 degrees flexion with manual maximum pull. In Group II, the initial tension of the PL bundle was intentionally higher than that of the AM bundle in every case at 30 degrees flexion, which was arthroscopically confirmed by probing after fixation. Group II was performed from October 1996 to October 2000 in 77 cases by TM.

In Group III, the PL bundle was first fixed to the anchor staple at 30 degrees flexion with 40 N using a spring-type tensiometer, and then the tension of the AM bundle was evaluated arthroscopically with a probe at 30 degrees. The initial setting force of the AM bundle which final tension was equal to that of the PL bundle was determined in 5 N increments. The AM bundle was fixed to the anchor staple at 30 degrees flexion with the determined force. Group III was performed from November 2000 to September 2002 in 48 cases by TM. In Group I and II, the initial force was not measured or determined by a tensiometer, but just the tension balance between AM and PL bundles was checked by probing after fixation.

The current clinical analysis included patients of unilateral ACL injuries operated with a double-bundle reconstruction and without any additional ligament surgery. Exclusion criteria was as follows: patients who underwent ACL reconstruction within one month after injury were excluded because acute phase surgery had been routinely refrained and only locked knees underwent ACL surgery combined with meniscus procedures within one month after injury; patients with significant radiographic osteoarthritic change with pain (two patients); two traumatic reinjury cases after 4 months and 15 months from the primary surgery, respectively; One female patient suffered from MRSA infection immediately after reconstruction, which resulted in residual abnormal instability. Detailed data of patients involved in the study are shown in Table 1. The three groups showed any statistical difference regarding demographic data. No patient was skeletally immature with a high intensity line for a T2-weighted MRI. The number of patients with accompanying complete meniscal injuries and full thickness cartilage injuries was shown in Table 2. The ACL reconstructions and postoperative management were performed in Tokyo Medical and Dental University Hospital and in one of its branch hospitals with the same protocol.

**Table 1:** Preoperative demographic data of each three group

Group	I	II	III
Number of patient	59	53	39

Average age at surgery (range)	26.8 (16-67)	27.5 (12 - 66)	24.0 (14-39)

Male - female ratio	24/35	23/30	21/18

Follow-up period (months)(range)	32.9 (24 - 60)	33.9 (24 - 60)	35.6 (24 - 61)

Preop. period (months)(range)	13.9 (2 - 40)	27.5 (1 - 192)	14.4 (1 - 180)

Preoperative Tegner score (range)	6.7 (4 - 8)	7.1 (4 - 9)	7.1 (3-9)

Every data were shown in average (range).

There was no significant difference among the three groups regarding preoperative demographic data.

**Table 2:** Accompanying injuries of each three group

Group	I	II	III
Number of patient	59	53	39

Medial meniscus injury	22	27	17

Resection	9	18	5

Repair	4	1	3

Lateral meniscus injury	16	17	10

Resection	4	11	4

Repair	4	0	0

Articular cartilage defect	1	2	0

### Operative Procedures

The operative procedures were performed using an arthroscopically-assisted technique for all groups. The procedure was performed using Acufex ACL Director Guide Systems (Smith & Nephew, Andover, MA). Two double-strand semitendinosus tendons were created in the majority of the cases. Gracilis tendon was additionally harvested in 6% of the cases, a total of 10 patients, when the semitendinosus tendon was shorter than 22 cm or found to be very thin with a diameter of less than 7 mm in a quadrupled fashion. Thicker half of the tendon was used for the AM bundle and the thinner half for the PL bundle. Recent anatomic study of the normal knee joint supports the selection of the graft thickness [15]. The graft was fixed for the femoral site with EndoButton^® ^(Smith & Nephew, Andover, MA). Tibial site was fixed to the suture post or anchor staple by a pull-out fixation method. Non-absorbable #5 and #2 sutures were used at both ends of the graft tissue. Two tibial guide wires were inserted in the tibial remnant tissue of the ACL. The wire for the AM bundle was aimed at the center of the remnant. The tibial guide wire for the PL bundle was aimed at the portion 3 or 4 mm posteriorly from the AM guide wire. Then, the tibial drill hole position was checked by a two-directional radiograph in full extension with the anterior wall of the intercondylar notch and intercondylar tubercles as radiographic landmarks [16,17]. The femoral drill hole position was at 11:30 for the AM bundle and at 10:30 for the PL bundle as "intercondylar clock" time for the right knee [18]. The center of the AM and PL tunnels were positioned about 6 mm anteriorly from the posterior margin between the intercondylar notch and the articular cartilage in flexion position. The graft tissue for the PL bundle was first inserted from the tibial tunnel into the joint, then through the femoral tunnel. The two graft tissues were fixed to the tibial post screw or anchor staple according to each fixation protocol of Group I - III

### Postoperative rehabilitation

The knee joint was immobilized in extension with a removable postoperative brace. Weight-bearing with 2 crutches and quadriceps setting were started 1 day after surgery. The amount of weight-bearing was increased gradually. Full weight-bearing gait was allowed with 2 crutches 4 weeks postoperatively. Knee range-of-motion exercise was started 3 days after surgery. Knee motion exercise was progressed as an active exercise to gain full extension and more than 90 degrees of flexion at 1 week and 120° at 4 weeks after surgery. Full recovery of knee motion was expected 3 months after surgery. Muscle strengthening was encouraged starting 6 weeks after surgery by the closed kinetic exercise. Running exercise was started at 3 months, first as jogging, and the running speed was gradually increased. Eighty percent of full-speed running was achieved, then athletic exercises related to the previous sports or desired sporting activities were initiated with detailed instructions. Athletic exercises were specific to each patient, depending on the kind of sports previously engaged in, as well as the patient's athletic level. Full athletic activities were allowed 6 months after surgery when sufficient muscle recovery after specified athletic training had been accomplished [19].

#### Clinical evaluation methods

The knee joint condition was evaluated on the basis of side-to-side differences between the injured and the uninjured legs. A full evaluation using our knee chart was performed every 3 months.

Each knee extension angle was measured by a goniometer with the leg passively raised. The differences of the knee extension angle were measured in one degree increment. The differences were confirmed by a lateral radiography of the knee in full extension [20]. Passive fully flexed knee angle was also measured with a goniometer for each leg. The differences of the knee flexion angle were measured in 5 degrees increments.

Manual knee laxity tests (Lachman test, anterior drawer test (ADT) and pivot-shift test) were performed and assessed for both legs. The differences between the injured and the uninjured legs were evaluated and categorized as negative, 1+, 2+ and 3+. The anterior knee laxity measured with the KT-1000 arthrometer (MedMetric, San Diego, CA) at manual maximum pull was expressed as the differences between the injured and the uninjured legs (KT measurements) [21]. The maximum extension and flexion strength of both knees were measured by Cybex machine (Lumex Inc, Ronkonkoma, NY) at 60 degrees/sec. The injured/uninjured limb differences were expressed as the percentage of the uninjured knee.

Lysholm knee scale was used as a general knee evaluation [22]. The subjective evaluation for the knee after surgery was expressed as a percentage of the uninjured knee as 100% [23]. With regard to sports activity level, performance and return to sporting activities and pre- and postoperative Tegner scores [24] were recorded. To determine the sports performance recovery score, the patients were asked how much their sporting activities had recovered in comparison to their pre-injury sports performance level of 100%.

Finally, the range of motion, KT measurements, knee extension strength at 60 degrees/sec by Cybex testing, the subjective assessment and the sports performance recovery score were categorized in accordance with the IKDC form [25,26]. In the IKDC form, category A indicates normal, category B indicates nearly normal, category C indicates abnormal and category D indicates severely abnormal. Practically, the classification was used as follows in this study: For the knee extension angle differences, category A is < 3 degrees, B is between 3 and 5 degrees, C is between 6 and 10 degrees, and D is > 10 degrees. For the knee flexion angle differences, category A is between 0 and 5 degrees, B is between 6 and 15 degrees, C is between 16 and 25 degrees, and D is > 25 degrees. For the KT measurements, category A is between - 2 mm < A < 3 mm, B is between -3 mm and - 2 mm or between 3 mm and 5 mm, C is -3 or less, or between 6 mm and 10 mm, and D is > 10 mm. For the knee extension strength evaluation at 60 degrees/sec, subjective recovery and sports performance recovery score, category A is 90% or more, B is between 89% and 76%, C is between 75% and 50%, and D is < 50% [1].

#### Statistical methods

Statistical analysis was performed using ANOVA for knee extension deficit, KT measurements, knee extension and flexion strength among the three groups. Fisher's PLSD test was used as a post hoc test. Kruskal-Wallis test was used for statistical analyses for pre- and postoperative Tegner scores, knee flexion deficit, manual knee laxity tests (Lachman test, ADT and pivot-shift test), total score of Lysholm knee scale, the subjective recovery assessment and the sports performance recovery score. Kruskal-Wallis test was also used for the subjective recovery scale, sports performance recovery scale, range of motion, KT measurements, and knee extension strength categorized in accordance with the modified IKDC evaluation among the three groups.

The significance was determined with a P value less than .05. A trend of significance was indicated when a P value was between .05 and .15. We used StatView for Windows (version 5.0) for the statistical evaluation (SAS Institute Inc., Cary, NC, USA).

## Results

Group I showed a significant extension deficit compared with groups II and III. There was a statistically significantly different among the three groups regarding Lachman test and pivot-shift test. Group III showed a trend of greater number of negative cases on two manual tests than the other groups. The average KT measurements were 2.1, 2.1 and 1.2 mm in Group I, II and III, respectively. ANOVA revealed a significant difference of anterior laxity measured by the KT-1000. The KT measurements of Group III were significantly smaller than that of Group I and II.

A trend of statistical difference was found among the three groups regarding Lysholm knee scale, subjective and sports performance recovery scales. Group I revealed significantly less score of Lysholm knee scale, subjective and sports performance recovery than Group II and III (Table 3). Each data was shown according to the modified IKDC category in Table 4.

**Table 3:** Summary of postoperative data of each three group

Group	I	II	III
Number of patient	59	53	39

Knee extension deficit (degrees) **	1.7 +/- 1.5	1.1 +/- 1.3	1.2 +/- 1.3

Knee flexion deficit (degrees)	1.4 +/- 2.8	1.1 +/- 2.5	0.9 +/- 1.8

Lachman test a)			

Negative	51	48	38

1+	6	5	1

2+	2	0	0

3+	0	0	0

Anterior drawer test			

Negative	55	50	36

1+	4	3	3

2+	0	0	0

3+	0	0	0

Pivot-shift test a)			

Negative	48	44	37

1+	10	8	2

2+	1	1	0

3+	0	0	0

KT measurements (mm) **	2.1 +/- 1.8	2.1 +/- 1.4	1.2 +/- 1.4

Knee extension strength (%)	92.3 +/- 19.5	90.5 +/- 15.6	94.7 +/- 10.2

Knee flexion strength (%)	91.3 +/- 18.9	94.2 +/- 12.5	94.8 +/- 11.5

Total score of Lysholm knee scale	92.1 +/- 7.1	94.0 +/- 9.1	94.8 +/- 4.9

Subjective recovery score b)	83.3 +/- 12.3	89.7 +/- 8.6	88.2+/- 10.4

Postoperative Tegner score	6.4 +/- 1.3	6.7 +/- 1.5	7.0 +/- 1.4

Sports performance recovery score b)	83.8 +/- 11.7	91.3 +/- 9.1	87.5 +/- 12.0

**: ANOVA indicated a statistically significant difference among the three groups.

a): Kruskal-Wallis test indicated a trend being statistically significantly different among the three groups.

b): Kruskal-Wallis indicated a statistically significant difference among the three groups.

**Table 4:** Summary of the data of each three group according to the IKDC category

Group		I	II	III
Patient number		59	53	39

Knee extension deficit	A b)	42	49	35

	B	17	1	4

	C	0	1	0

	D	0	2	0

Knee flexion deficit	A	58	49	39

	B	1	2	0

	C	0	0	0

	D	0	2	0

KT measurements	A a)	38	32	32

	B	18	21	7

	C	3	0	0

	D	0	2	0

Knee extension strength	A	34	34	27

	B	15	11	11

	C	8	5	0

	D	1	0	0

Not recorded		1	3	1

Subjective recovery score	A b)	28	36	25

	B	13	11	8

	C	16	5	6

	D	0	0	0

Not recorded		2	1	0

Sports performance recovery score	A b)	23	33	21

	B	19	9	12

	C	10	5	5

	D	0	0	0

Not recorded		7	6	1

a): Kruskal-Wallis test indicated a trend being statistically significantly different among the three groups.

b): Kruskal-Wallis test indicated a statistically significant difference among the three groups.

## Discussion

The current clinical study investigated the effects of different initial tensioning strategy on the outcome of DB ACL reconstruction. The study is the first investigation to evaluate the clinical differences caused by different initial tensioning in DB reconstruction. The study indicated that Group I (AM bundle fixed at manual maximum pull after PL bundle fixation at 30 degrees flexion) had the worse outcome among the groups. The AM bundle is thought to be more affected by the bony structure of the joint in extension position. Also, the outcome differences among the three groups suggest that unbalanced initial tension between the AM and PL bundles in DB ACL reconstruction is not recommended to achieve a better clinical outcome.

In-vitro cadaveric knee studies have raised concerns of high initial tension of the PL bundle in DB ACL reconstruction. Miura and Woo et al. demonstrated that equal tensioning of both the AM and PL bundles at 30 degrees flexion resulted in too high of a tension of the PL bundle. Graft fixation at 60 degrees flexion for the AM bundle and full extension for the PL bundle caused too much tension of the AM bundle [4]. The tension balancing in the DB ACL reconstruction seems not easily achieved. Higher initial tension of the AM bundle seems to cause extension deficit in some cases and cause graft failure in other cases.

With regard to the effect of initial tension magnitude in DB ACL reconstruction, Hoshino et al. demonstrated that a total of 50 N of tension force was assumed to be excessive for normalizing knee kinematics at a low flexion angle even if double bundle reconstruction was used. It was the case both when 50 N was applied to the AM bundle with 0 N on the PL bundle, and when 50 N was applied to the PL bundle with 0 N on the AM bundle [5]. Vercillo and Woo et al. recommended that the AM bundle should be fixed between 15 and 45 degrees flexion and the PL bundle should be fixed at 15 degrees to avoid hyper-strain on the knee joint [6].

However, the results above were from a time-0 cadaveric studies. Soft tissue creep and biologic changes after soft tissue implantation could not maintain the initial force of the graft tissue. The clinical significance of the results and recommendation from in vitro 0-time studies have not been clarified yet. Graft tissue is affected by stress relaxation, and the initial tension cannot be maintained after grafting. Especially in hamstring reconstruction, the initial tension would decrease more rapidly by stress relaxation compared with that in BPTB reconstruction [27]. Moreover, the grafted tissue changes in terms of its biologic and biomechanical character during the healing process. More complex effects are assumed in each AM and PL bundle in DB reconstruction. Previous reports suggest that hamstring ACL reconstruction is affected by initial tensioning to some degree [9], while BPTB ACL reconstruction is less affected by initial tension [10]. The results of the current study suggest that the difference of initial tension balance in each bundle affects the clinical outcome in DB ACL reconstruction.

Limitations of the current study are based on that the study was performed many years ago when graft position of the ACL surgery was done on the "isometric" concept avoiding notch impingement. The initial tensioning was performed manually in Group I and II. In Group III, it was set at 40 N with a spring-type tensiometer. The force setting itself might make some bias in the study. Future studies would be needed to evaluate whether the recommendation of the study is directly translated to "anatomic" DB ACL reconstruction with an accurate force setting instrument. Also, a randomized clinical trial will be necessary to elucidate the clinical advantages by the initial force setting attempting both AM and PL bundles equally at 30 degrees flexion.

## Conclusions

The current clinical study does not recommend manual maximum of initial tension applied to the anteromedial or posterolateral bundles with graft tension imbalance at 30 degrees of flexion in double-bundle ACL reconstruction to achieve a better clinical outcome.

## Competing interests

The authors declare that they have no competing interests.

## Authors' contributions

TM, KY and IS are the surgical team member who performed each surgery. TM is the chief of the group and performed drafting the manuscript. HK and YJJ helped to completing the manuscript with statistical analysis, designing and data base creation.

The authors approved the manuscript.
